# Impact of respiratory muscle training on exercise tolerance in hypobaric hypoxia: A pilot study in adult males

**DOI:** 10.14814/phy2.70613

**Published:** 2025-10-27

**Authors:** Scott K. Ferguson, Michael Miller, Luke Yeager, Annie Wislowski, Andrew Subudhi, Robert C. Roach

**Affiliations:** ^1^ Integrative Aerospace and Exercise Physiology Laboratory (IAEP), Department of Human Factors College of Arts and Sciences, Embry‐Riddle Aeronautical University Daytona Beach Florida USA; ^2^ Altitude Research Center, Department of Medicine, Division of Pulmonary Sciences and Critical Care Medicine University of Colorado Aurora Colorado USA; ^3^ Department of Human Physiology and Nutrition William J Hybl Sports Medicine and Performance Center, University of Colorado Colorado Springs Colorado USA

**Keywords:** exercise performance, exercise tolerance, hypobaric hypoxia, simulated altitude, V̇O_2_max

## Abstract

Hypoxia and certain chronic diseases can increase the work of breathing, potentially impacting the muscle hyperemic response during exercise and overall exercise performance. Respiratory muscle training (RMT) has been observed to decrease perceived effort under hypoxic conditions; however, it remains unclear if RMT affects exercise performance in acute hypoxia. We investigated whether 4 weeks of RMT could enhance submaximal and maximal exercise performance in hypoxia (barometric pressure = 425 mmHg, ~4876 m). Seven adult males underwent a 250 kJ submaximal (target RPE 15–17) cycling trial in hypoxia, followed by an incremental test to maximal exertion. After 4 weeks of RMT under normoxic conditions, these tests were repeated in hypoxia. After RMT, RPE showed no significant difference during the submaximal test (Pre‐RMT: 16 ± 0.45, Post‐RMT: 16 ± 0.45, *p* > 0.05) despite an increase in HR (Pre‐RMT:154 ± 16, Post‐RMT: 161 ± 12, *p* < 0.05). There were no significant differences in submaximal exercise performance. However, RMT did raise maximal oxygen uptake (V̇O_2_max) peak power (Pre‐RMT: 243 ± 35, Post‐RMT: 252 ± 38 W, *p* = 0.04, Cohen's dz. = 0.97). Future investigations should explore the potential effects of RMT on perceived exertion and exercise tolerance at altitude. The results of this pilot study suggest that RMT may improve peak power at V̇O_2_max, however given the limited sample size of this investigation, further research is required to determine if RMT impacts performance in other domains of exercise intensity. Furthermore, whether RMT impacts performance at altitude in females remains unknown.

## 1 INTRODUCTION

The metabolic demands of strenuous exercise require a sufficient supply of oxygen (O_2_), which is provided by a coordinated effort of the cardiovascular and pulmonary systems (Joyner & Wilkins, 2007). Despite the increased demand for O_2_ (V̇O_2_) by locomotor muscles, exercise at these higher intensities (i.e., within the severe‐intensity domain) also results in a non‐linear increase in total ventilation (V_E_), increasing the work of breathing (W_b_) and oxygen cost of respiration (up to 15% of total V̇O_2_ in highly fit subjects (Aaron et al., 1992)). The greater respiratory muscle V̇O_2_ has been associated with a redistribution of cardiac output of up to 16% (Harms et al., 1998), which is diverted from the locomotor muscles via sympathetically mediated vasoconstriction and sent to support the work requirements of the respiratory muscles (Harms et al., 1997).

Rapid ascent to high altitudes and exposure to simulated hypoxia also causes a rapid increase in V_E_, which helps to offset hypoxemia and preserve the aerobic work capacity of lowlanders (West, 2000). However, the hypoxia‐induced elevation in W_b_ is exacerbated during heavy and severe intensity exercise (~70% V̇O_2_max, (Fitting, 1991)) and further challenges the cardiorespiratory and locomotor systems and work capabilities in these low partial pressure of oxygen (PO_2_) environments (Vogiatzis et al., 2008). Many patient populations suffering from systemic diseases that increase *W*_b_ (e.g., chronic heart failure) may also experience a blood flow “stealing” effect of the respiratory muscles, which contributes to the reduced exercise capacity emblematic of these illnesses (Miller et al., 2007; Musch, 1993; Smith et al., 2018). This phenomenon is partly driven by the respiratory muscle metaboreflex—a reflex arc activated by the accumulation of fatigue‐related metabolites in the respiratory muscles, which triggers sympathetic vasoconstriction and redistributes blood flow away from limb muscles to support the fatigued respiratory muscles (Witt et al., 2007). To combat these challenges, several investigators have used respiratory muscle training (RMT) to attenuate the accumulation of fatigue‐associated metabolites, which activate phrenic afferents and increase sympathetic vasoconstrictor activity in working skeletal muscle (Witt et al., 2007). RMT may indirectly improve blood distribution to limb locomotor muscles during exercise and, thus, may improve exercise capacity (McConnell & Romer, 2004).

While there have been many investigations into the effects of RMT on exercise performance in normoxia (reviewed by (Illi et al., 2012a)), less attention has been given to its potential to improve exercise performance in hypoxia, with some (Downey et al., 2007; Helfer et al., 2016; Keramidas et al., 2011; Lomax et al., 2017) but not all (Esposito et al., 2010) studies showing beneficial effects of RMT on exercise performed in normobaric or hypobaric hypoxia. Importantly, the ergogenic effects of RMT have also been associated with changes in perception of exertion during exercise (Keramidas et al., 2011; Lomax et al., 2017), which plays a vital role in exercise performance in hypoxia (Noakes, 2004). Thus, RMT may reduce the perceived effort of exercise intensity at the limit of sustainable work rates (e.g., at or slightly below critical power). This idea is supported by Downey et al. (2007), who reported the ratings of perceived exertion (RPE) and dyspneic sensations during exercise following resistance RMT, which were significantly decreased in the RMT group.

Despite the prospect of RMT to improve exercise performance and alter the perception of exertion during heavy‐intensity exercise in hypoxia, to our knowledge, there have been no investigations into the effects of endurance RMT on a self‐paced (i.e., RPE‐based) measure of exercise performance during acute exposure to hypobaric hypoxia. Thus, the purpose of this investigation was to evaluate the effects of 4 weeks of endurance respiratory muscle training (RMT) using eucapnic voluntary hyperventilation on exercise performance during acute exposure to simulated high altitude (4876 m). We tested the hypothesis that RMT would improve exercise performance in hypoxia, as evidenced by greater work output during a self‐paced 250 kJ submaximal cycling trial targeting a rating of perceived exertion (RPE) between 15 and 17, as well as enhanced performance during an incremental cycling test to maximal exertion.

## 2 METHODS

### 2.1 Subjects and ethical approval

A total of 7 healthy, recreationally active male adults completed the study (Age: 28.5 ± 6.3 yrs, height: 182.25 ± 6.84 cm, weight: 77.25 ± 7.35 kg, body mass index: 23.91 ± 1.66 kg/m^2^). Subjects aged 18–45 years old, able to achieve a power output ≥3.5 W/kg body weight during an incremental exercise to maximal exertion on a cycle ergometer, and free of known disease were included in the study population. Subjects were excluded if they reported a history of recent altitude exposure >1650 m (including air travel in the past 3 weeks), medications or dietary supplements, smoking, or conditions known to affect O_2_ transport and/or performance at high altitude. For the duration of the study, subjects were required to avoid travel, keep a log of their aerobic exercises outside of the study requirements, and maintain a consistent fitness level throughout the study. Before entering the study, all participants provided written consent, and all protocols utilized herein were approved by the Colorado Multiple Institutional Review Board (protocol number 18‐0464).

### 2.2 Study overview

**FIGURE 1:**
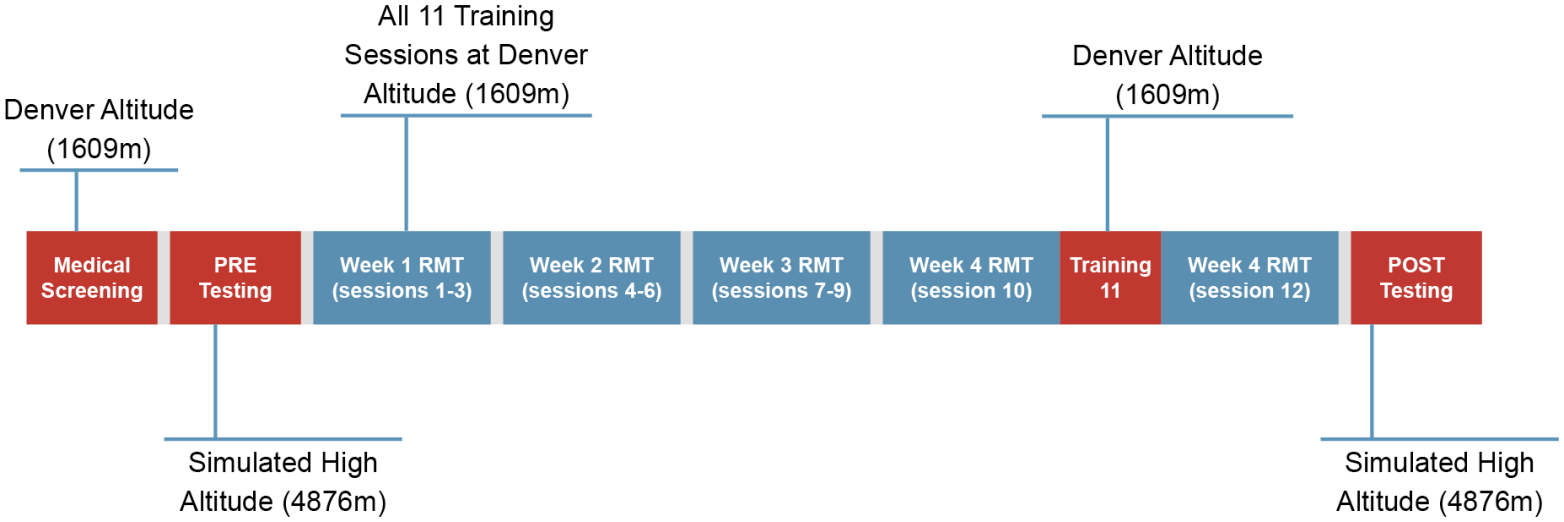
Experimental procedures and test schedule. After pre‐screening, participants completed Pre‐RMT exercise testing in a hypobaric chamber (4876 m), followed by 4 weeks of endurance RMT (30 min/session, 3 sessions/week). After a 1‐week recovery period, participants completed Post‐RMT testing in the hypobaric chamber (4876 m).

Figure 1 provides an overview of the experimental procedures and test scheduling. Participants visited the laboratory a total of 15 times throughout the duration of the study. Exercise testing was performed during 4 periods that included an initial medical screening session, Pre‐RMT, second medical assessment, and Post‐RMT testing. The medical screening and medical assessment testing were completed at low altitudes under ambient conditions (Barometric pressure = 625 mmHg,1650 m), while the Pre‐ and Post‐RMT testing was performed at a simulated high altitude over a ~ 6‐h exposure period in a hypobaric chamber. The chamber was decompressed to 425 mmHg for the simulated altitude exposure, resulting in an inspired PO_2_ (PiO_2_) of 79 mmHg (~4876 m). Primary outcome measures were time to complete a self‐paced 250 kJ submaximal cycling exercise test (submax‐CT), targeting an RPE of 15–17, and an incremental test to maximal exertion for the determination of maximal oxygen uptake (V̇O_2_max).

Between Pre‐ and Post‐RMT testing, participants completed 11 sessions of endurance RMT over the course of 4 weeks (see RMT section below). During the fourth week, between RMT sessions 10 and 11, subjects completed an additional medical assessment, submax‐CT, and V̇O_2_max test at low altitude (1650 m). This second medical assessment was designed to verify that subjects still met the initial performance inclusion criteria and to refamiliarize them with the protocol before completing the Post‐RMT testing in hypoxia. After completion of the 11th and final RMT training session, participants took a week of recovery prior to completing the Post‐RMT testing at high altitude.

### 2.3 Respiratory muscle training

All RMT training was accomplished using a custom‐built device (Olin et al., 2012). The device allowed staff to ensure that subjects remained eucapnic during training and adhered to predefined respiratory rate and minute ventilation targets throughout the training. Subjects were seated in front of a metronome and monitors displaying real‐time minute ventilation and breaths per minute, which allowed subjects to match their targets. To achieve an optimal training volume, required minute ventilation values were calculated using 55% of the subject's slow vital capacity (SVC) as determined during the medical screening and described by Leddy et al. (2007). Subjects were informed of these values prior to and throughout each RMT session.

Subjects underwent 11, 30‐min sessions of RMT over the course of 4 weeks at baseline altitude (1650 m). Each week consisted of 3 training sessions, with the final week consisting of 2 training sessions and a testing session (T11) between training sessions. Each subject was started at a baseline rate of 30 breaths per minute on day one of training. After 20 min of maintaining this rate with the previously calculated minute ventilation, subjects were then instructed to increase their rate by 2 breaths per minute for the final 10 min of training. The next training session began at the highest respiratory rate achieved during the previous session. Each subject's minute ventilation was then adjusted each session to match their target respiratory rates. Previous investigations have supported the use of a 4‐week RMT program to induce significant improvements in performance (Notter et al., 2023).

### 2.4 Exercise testing

All participants were familiarized with the equipment and the RPE scale prior to testing. The Borg 6–20 scale was used to quantify perceived exertion, where 6 corresponds to “no exertion at all” and 20 corresponds to “maximal exertion (Borg, 1982).” Participants were instructed that the RPE scale represents their subjective perception of effort, taking into account sensations of breathing, leg fatigue, and overall exertion, and were encouraged to use the full range of the scale when appropriate. All exercise testing was completed in ambient air on a computer‐controlled electromagnetically braked cycle ergometer (Velotron, Elite Model, Racer Mate, Seattle, WA, USA) for the determination of revolutions per minute (RPM) and time (s). O_2_ saturation (*S*pO_2_) and heart rate (HR) were measured using a pulse oximeter (Nellcor N‐595, Pleasanton, CA, USA) with adhesive forehead sensors and a three‐lead ECG (ADI Instruments ML132 BioAmp, ADI Instruments, Bella Vista, Australia), respectively. Pulmonary gas (O_2_ and CO_2_) measurements were measured through a dual O_2_ and CO_2_ sensor (Oxigraf O_2_Cap Oxygen Analyzer, Oxigraf, Sunnyvale, CA, USA) sampled from a 3‐L mixing chamber. Ventilation was measured using a turbine spirometer (UVM Vacumed, Ventura, CA, USA) on the outflow track of the mixing chamber and corrected for temperature, humidity, and barometric pressure in the mixing chamber (ADI Instruments, Thermistor Pod, Colorado Springs, CO, USA).

### 2.5 Self‐regulated, submaximal cycling trial

Participants completed a 5‐min warmup at an RPE of 9. For the submax‐CT, participants were instructed to complete the 250 kJ ride while pacing their effort according to RPE. Participants had control over their individual resistance (W) but were blinded to the W they had selected. Participants were encouraged to maintain RPE between 15 and 17 and were aware that the effort was timed, but instructed to maintain a submaximal effort. The goal of maintaining an RPE between 15 and 17 was aimed at keeping the participant near the top‐end of their heavy‐intensity domain. At the 50% (125 kJ) and 100% (250 kJ) intervals, measurements of V̇O_2_, respiratory exchange ratio (RER), ventilatory equivalent for carbon dioxide (V̇E/V̇CO_2_), and minute ventilation (V̇E) were recorded. Following completion of the trial, participants were given a 20‐minute recovery before beginning the incremental V̇O_2_ max test. During the Pre‐ and Post‐RMT testing days, the submax‐CT was initiated after 4.5 h at simulated high‐altitude (4876 m).

### 2.6 Incremental exercise test

After adjusting the ergometer and fitting the subjects with the gas collection mask, 1–2 min of resting data were collected to ensure signal integrity. The incremental test was then initiated at 25 W, and the workload was increased 25 W/min to volitional exhaustion or until the subject was unable to maintain a 50 RPM cadence while remaining seated on the cycle ergometer. Measurements of RPE, HR, SpO_2_, and RPM were taken during the last 15 s of each 25 W step. The V̇O_2_max was defined as the highest V̇O_2_ obtained over a 20 s averaging period and peak power output was determined as the highest power output maintained for 1 min.

### 2.7 Validation of the V̇O_2_Max

Following the incremental ramp test, participants remained on the bike, and V̇O_2_ max validation was completed via a step protocol, which included 8 min of riding at 25–100 W (participant chose the specific resistance), 2 min of riding at 33% validation wattage (V̇O_2_ max wattage +25 W), 30 s at 50% validation wattage, and finally, 100% validation wattage until participant voluntary exhaustion or RPM < 50. This validation protocol follows recommendations from Poole and Jones (Poole & Jones, 2017) and provides a reliable means to distinguish V̇O_2_max from V̇O_2_peak.

### 2.8 Statistical analysis

All between‐group (Pre‐ vs. Post‐RMT) comparisons were made using paired one‐tailed Student's *t*‐tests, with the exception of SaO_2_, which was compared using a one‐way analysis of variance (ANOVA) with repeated measures. Data analysis was performed using GraphPad Prism version 9.3.1 (Dotmatics, San Diego, CA, USA). Statistical significance was set at *p* < 0.05 and all values are expressed as means ± standard deviation of the mean (SD). Given the exploratory nature of this study and the small sample size (*n* = 7), paired *t*‐tests were chosen to compare pre‐ versus post‐RMT changes in mean and peak values. While additional time‐point data were collected during the submaximal cycling test, we intentionally limited our analysis to summary statistics to minimize the risk of Type I error associated with repeated comparisons. Moreover, because the self‐paced nature of the submaximal test resulted in variable trial durations and pacing strategies across participants, aligning discrete time‐points for group‐level repeated measures ANOVA presented substantial interpretational limitations.

## 3 RESULTS

### 3.1 Baseline measurements and subject compliance

**FIGURE 2:**
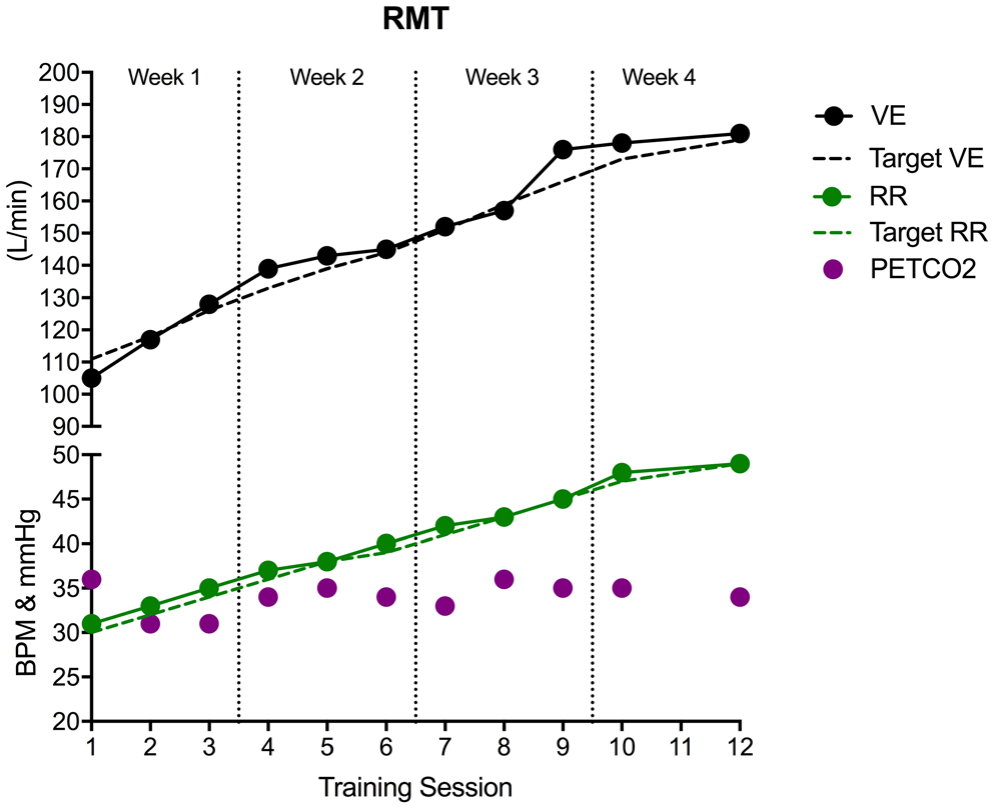
Participant progression through 12 endurance RMT sessions. Training began at 30 breaths/min on Day 1. After 20 min at this rate using the prescribed minute ventilation, participants increased breathing rate by 2 breaths/min for the final 10 min. Each subsequent session began at the highest rate achieved previously. PETCO_2_, end‐tidal partial pressure of CO_2_; RR, respiratory rate; V̇E, ventilation.

Figure 2 shows the progression of a representative subject through 4 weeks (11 sessions) of the RMT training protocol. All subjects (*n* = 7) successfully completed the Pre‐RMT testing, RMT training protocol, and Post‐RMT testing sessions, and there were no differences in Pre‐ versus Post‐RMT SVC (Pre‐RMT: 5.36 ± 0.56, Post‐RMT: 5.39 ± 0.78 L, *p* = 0.77).

### 3.2 Effect of RMT on submax‐CT performance and ventilatory efficiency during submaximal exercise

**FIGURE 3:**
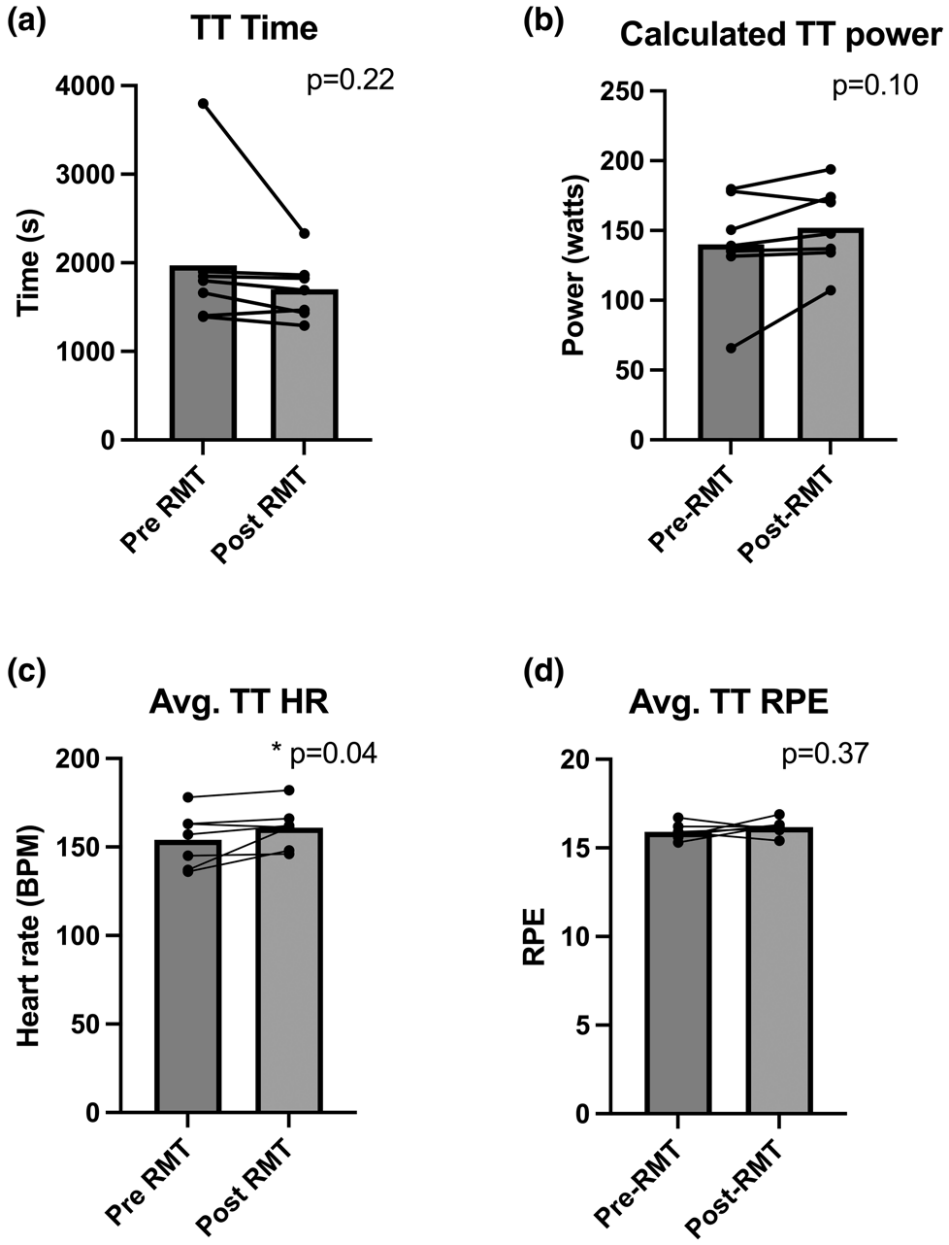
Performance during a self‐paced 250 kJ submax‐CT. (a) Total time; (b) Average power; (c) Average heart rate; (d) Average RPE. Data analyzed using paired one‐tailed Student's *t*‐tests (*n* = 7 males). BPM, beats per minute; CT, cycling trial; HR, heart rate; RPE, rating of perceived exertion; submax‐CT, submaximal cycling trial.

**FIGURE 4:**
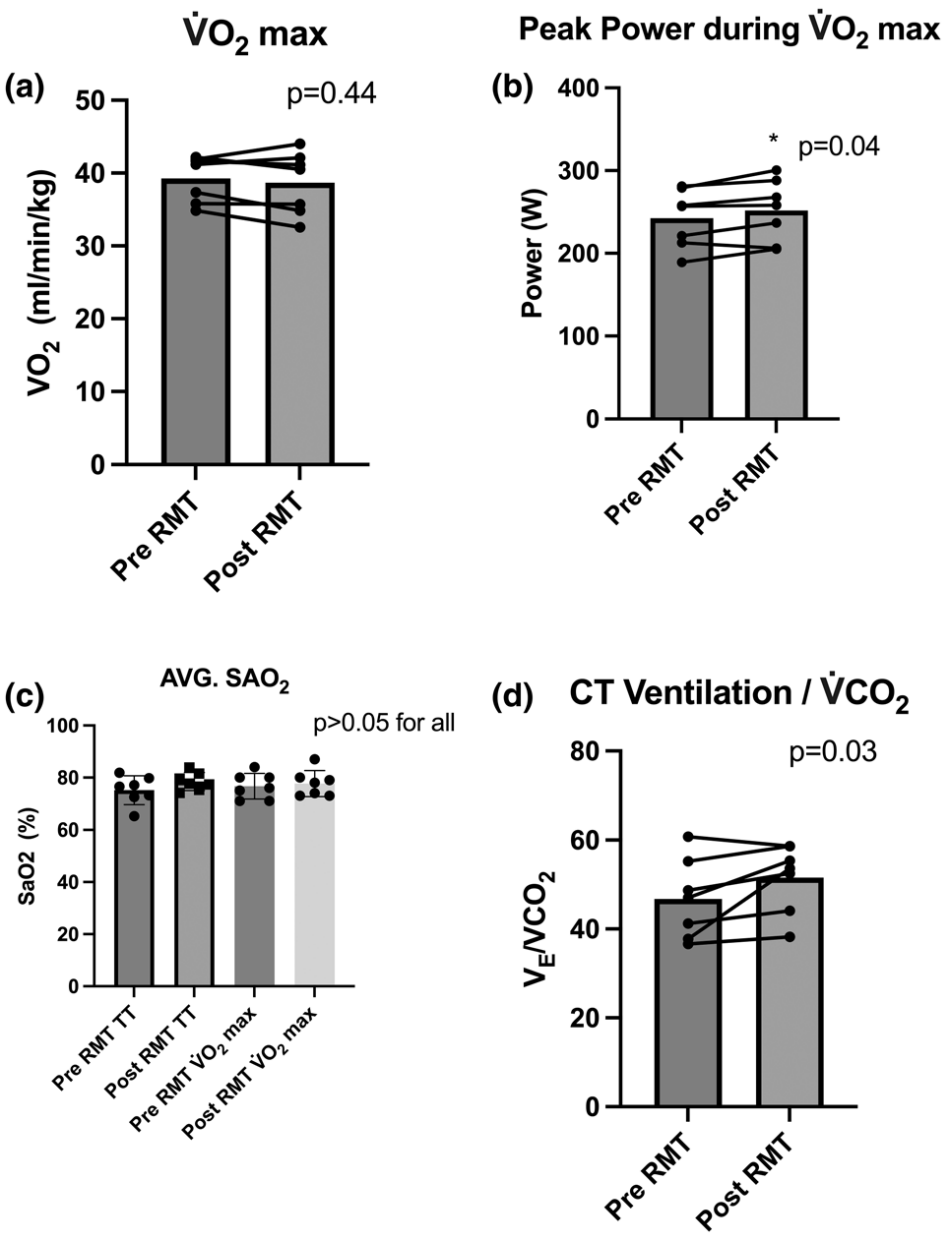
Pre‐ versus post‐RMT metabolic and power responses to maximal exercise testing in simulated altitude (4876 m). (a) Relative V̇O_2_max values measured Pre‐ and Post‐RMT, (b) Total ventilation to CO_2_ exhalation ratio measured during the submax‐CT, (c) Average O_2_ saturation of blood measured during the submax‐CT and V̇O_2_ max test using a Nellcor pulse oximeter, (d) Pre‐ versus Post‐RMT V̇O_2_max in normoxia, Pre‐ versus Post‐RMT peak power obtained during V̇O_2_max test in hypoxia, Pre‐ versus Post‐RMT peak power obtained during V̇O_2_max test in normoxia (*n* = 7 males for each panel). Data from Panels A, B, D, E, and F were compared using paired one‐tailed Student's *t*‐tests. Data from panel C were compared using a one‐way analysis of variance (ANOVA) with repeated measures. V̇CO_2_, volume of expired CO_2_; V̇_E_: total ventilation; V̇O_2_max, maximal oxygen uptake; SAO_2_, arterial oxygen saturation.

Table 1 shows the Pre‐ versus Post‐RMT pulmonary SVC (measured prior to testing) and metabolic parameters measured during the submax‐CT. There were no significant Pre‐ versus Post‐RMT differences in SVC, V_E_, V̇O_2_, V̇CO_2_, RER, or respiratory rate during the trial (>0.05 for all). Figure 3 shows the average time to complete the trial (A), average power output (B), and average HR and RPE (C and D). Despite a 13% decrease in time to completion, the effect of RMT on this parameter failed to reach statistical significance (Pre‐RMT: 1971 ± 832, Post‐RMT: 1701 ± 349 s, *p* = 0.22; Cohen's dz. = −0.51 Figure 3a). Additionally, there were no significant differences in average submax‐CT power output (Pre‐RMT: 140 ± 38, Post‐RMT: 152.1 ± 29 W, *p* = 0.10; Cohen's dz. = 0.73, Figure 3b). Participants had a significantly higher average Post‐RMT HR when compared to Pre‐RMT testing (Pre‐RMT:154 ± 16, Post‐RMT: 161 ± 12, *p* = 0.04, Cohen's dz. = 0.77, Figure 3c). As instructed, there was no significant difference in average submax‐CT RPE (*p* = 0.19, Figure 3d). There was also no significant difference in Pre‐ versus Post‐RMT mean SaO_2_ during the submax‐CT (*p* > 0.05, Figure 4c). During the final 10% of the submax‐CT in hypoxia, the V̇E/V̇CO_2_ ratio, was significantly greater following RMT (*p* = 0.03, Figure 4b), with all but one subject increasing their values when compared to Pre‐RMT.

**TABLE 1:** Pre‐ versus post‐RMT pulmonary slow vital capacity and metabolic parameters measured during the final 10% of a self‐paced 250 KJ cycling test.

	Pre‐RMT (*n* = 7)	Post‐RMT (*n* = 7)	*p*‐value
SVC (L)	5.36 ± 0.56	5.39 ± 0.78	>0.05
V̇E (L/min, BTPS)	96.3 ± 44	118.0 ± 30	>0.05
V̇CO_2_ (L/min)	1.98 ± 0.56	2.27 ± 0.38	>0.05
V̇O_2_ (L/min)	2.17 ± 0.48	2.52 ± 0.41	>0.05
RER	0.91 ± 0.08	0.90 ± 0.06	>0.05
RR (BPM)	43.2 ± 28.2	44.8 ± 20.3	>0.05
Vᴛ (L/breath)^a^	2.40 ± 0.63	2.82 ± 0.64	>0.05

*Note*: All exercise tests were performed at a simulated altitude of 4876 m. Values were compared using paired Student's *t*‐tests (*n* = 7 males).

^a^ Vᴛ (tidal volume) derived as V̇E (BTPS)/RR; expressed as L/breath.

### 3.3 Effect of RMT on incremental exercise test

There were no significant differences in Pre‐ versus Post‐RMT V̇O_2_max, V̇CO_2_, RER, V̇_E_, HR, and final RPE obtained during the V̇O_2_max test (Table 2, *p* > 0.05 for all). None of the V̇O_2_max validation trials resulted in a greater V̇O_2_ than the incremental exercise test. Four weeks of RMT resulted in no significant differences in V̇O_2_max during exposure to hypoxia (Figure 4a). However, RMT resulted in a 4% increase in V̇O_2_max peak power (Pre‐RMT: 243 ± 35, Post‐RMT: 252 ± 38 W, *p* = 0.04, Cohen's dz. = 0.97, Figure 4e). To explore whether changes in relative aerobic intensity influenced performance, we assessed the relationship between the change in %V̇O_2_max during the submaximal cycling trial and the change in time to completion from Pre‐ to Post‐RMT and found no significant relationship (*r* = 0.03, *p* = 0.95).

**TABLE 2:** Pre‐ versus Post‐RMT maximal oxygen uptake (V̇O_2_max) test results.

	V̇O_2_ (L/min)	V̇CO_2_ (L/min)	RER	V̇_E_ (L/min)	Vᴛ (L/breath)^a^	HR (BPM)	Final RPE
Pre‐RMT	2.86 ± 0.3	3.28 ± 0.5	1.14 ± 0.1	154.87 ± 33	2.73 ± 0.7	165 ± 10	19 ± 1
Post‐RMT	2.92 ± 0.3	3.36 ± 0.6	1.15 ± 0.1	159.04 ± 24	2.92 ± 0.75	167 ± 11	19 ± 1

*Note*: All exercise tests were performed at a simulated altitude of 4876 m. Values were compared using paired one‐tailed Student's *t*‐tests (*n* = 7 males), *p* > 0.05 for all.

^a^ Vᴛ (tidal volume) derived as V̇E (BTPS)/RR; expressed as L/breath.

## 4 DISCUSSION

The principal original finding of this investigation was that 4 weeks of endurance RMT did not significantly increase submax‐CT performance or V̇O_2_ max in simulated high‐altitude (4876 m). While these findings contradict our original hypothesis, RMT did increase peak power output achieved during the incremental exercise test to exhaustion (V̇O_2_ max test). In the present study, HR was higher post‐RMT at a fixed RPE during the submaximal trial, while power output remained unchanged. This finding does not indicate enhanced performance; rather, it reflects a modest shift in the HR–RPE relationship. Given the small sample size and the inherent day‐to‐day variability in HR, we interpret this cautiously and do not consider it evidence of improved tolerance. However, the increased power output achieved during the incremental exercise test does warrant further investigation into the effects of RMT on exercise performance in hypoxia.

### 4.1 Relationship to the existing literature

The study's main finding was that 4 weeks of endurance RMT did not impact the performance of a self‐paced 250 KJ cycling test or V̇O_2_ max in simulated (hypobaric) high‐altitude (4876 m). Investigations into the impacts of RMT on exercise performance have traditionally focused on normoxic environments (Illi et al., 2012b), and while some findings have shown ergogenic effects in hypoxia (Downey et al., 2007; Helfer et al., 2016; Keramidas et al., 2011; Lomax et al., 2017), not all investigations have shown positive results (Esposito et al., 2010). A defining feature of the present investigation is the use of a self‐paced 250 KJ cycling test, which affords the measurement of exercise performance at the top of the subjects' perceived heavy‐intensity domain (e.g., RPE range of 15–17). However, while six out of seven participants showed numerical improvements in test performance, trial time was not significantly faster following RMT. In this regard, it's crucial to acknowledge the sensitivity of submaximal exercise testing (Rienks et al., 2015; Williams et al., 2019). The high likelihood of day‐to‐day variability could have contributed to the difficulty in detecting differences, if they existed, despite the 4 weeks of RMT intervention. This variability in performance outcomes underscores the complexity of assessing the efficacy of RMT, especially in hypoxic conditions, where physiological responses may vary considerably among individuals (Fulco et al., 2013). Thus, future studies exploring the effects of RMT on exercise performance should consider incorporating measures to account for day‐to‐day variability and further elucidate the mechanisms underlying its potential ergogenic effects in hypoxic environments.

From a physiological perspective, while RMT could have augmented blood flow distribution during exercise (i.e., reduced the stealing effect of the respiratory muscles), the level of hypoxia utilized in the present investigation may have prevented the higher average power output from significantly reducing submaximal trial time. More specifically, the limitations of exercise performance in hypoxia have been explored by many investigations. In 1996, Wagner (Wagner, 1996) used a model to explore how changes in O_2_ delivery may (or may not) impact V̇O_2_max at extreme altitudes. Due to the diffusive limitations associated with high elevations (e.g., reduced microvascular partial pressure of O_2_ (PO_2_) and, thus, muscle O_2_ diffusion capacity (D_m_O_2_)), alterations in bulk blood flow afforded by RMT may have only slightly altered performance in this extreme environment. Despite this notion, the significantly greater power output obtained during the V̇O_2_max test and greater HR during the submax‐CT (without a difference in RPE) suggests that RMT may offer beneficial adaptations for those exercising in hypoxic conditions (e.g., enhanced tolerance to dyspnea/exertional breathing).

In a similar report by Keramidas, Kounalakis and Mekjavic (Keramidas et al., 2011), 4 weeks of endurance RMT (5 days a week) resulted in a significant increase in V̇O_2_max in hypoxia, despite no improvements in a constant load exercise test in normobaric hypoxia. The findings of the present investigation contrast with this report, which could be due to differences in experimental design. While normobaric hypoxia has long been used as an altitude mimetic, some differences may alter the *W*_b_ to a greater extent in normobaric hypoxia when compared to hypobaric hypoxia. More specifically, Loeppky et al. (1997) demonstrated that *V*_E_ was 39% greater after 3 h of exposure to normobaric versus hypobaric hypoxia (later corroborated by Savourey et al. (2003)). Given the greater total ventilation and air density of normobaric versus hypobaric hypoxia, the elevated V̇O_2_max observed following RMT by Keramidas et al. (2011) could be the result of higher Wb, which necessitated an increased V̇O_2_ of the respiratory muscles. In other words, the effects of RMT in normobaric hypoxia could have manifested as increased V̇O_2_ of the respiratory muscles rather than altered hemodynamic responses to exercise, which may increase exercise capacity. The elevated peak power output observed during V̇O_2_max testing, despite unchanged whole‐body V̇O_2_ and V̇_E_, suggests that RMT may have induced physiological adaptations that reduced the metabolic demands of the respiratory muscles. This could have resulted in a redistribution of cardiac output toward the locomotor muscles during high‐intensity exercise, as previously demonstrated in studies showing reduced respiratory muscle metaboreflex activity following RMT (Harms, 2007; Harms et al., 1997; Witt et al., 2007). These changes likely enhanced the mechanical output achievable during exercise in hypoxia without necessitating a corresponding increase in O_2_ consumption.

In the present investigation, the RMT‐induced increases in power during the V̇ O_2_max test in the absence of altered V̇O_2_max may reflect a small but significant reduction in the metabolic perturbation of the respiratory muscles. These changes would be expected to increase the blood flow available to the locomotory muscles (Harms et al., 1997). Despite these prospects, further investigation into how endurance‐RMT alters blood flow distribution and heterogeneity during exercise in hypoxia is warranted.

### 4.2 Applied and clinical relevance

These findings suggest that RMT may enhance exercise capacity during maximal exertion, which is relevant for military personnel and individuals working at high altitudes. Additionally, conditions such as chronic heart failure (CHF) and COPD increase the Wb and impair respiratory function, contributing to exercise intolerance (Harms, 2007; Mancini et al., 1995; Weiner et al., 1999). RMT has been shown to improve respiratory muscle endurance and perceived exertion in these populations (Mancini et al., 1995; McConnell et al., 2003; Weiner et al., 1999). The elevated HR at a fixed RPE observed here aligns with prior findings that RMT may influence perceptual responses to exercise (Downey et al., 2007). Notably, hypobaric hypoxia shares physiological features with CHF and COPD, including reduced O_2_ diffusion capacity and increased Wb. These parallels highlight the potential for RMT to serve as a non‐invasive intervention in both clinical and extreme environmental settings. Future studies should explore these effects in more diverse populations, including females and those with chronic disease.

### 4.3 Experimental considerations and future directions

The current study employed isocapnic hyperpnea as an endurance training method rather than resistance RMT, which is commonly available commercially. This approach may have yielded different results than resistance RMT devices. Therefore, further research should explore the effects of endurance versus resistance RMT on exercise capacity and perceived exertion in hypoxic conditions.

A clear weakness of the present investigation is the small sample size, with only seven adult male volunteers, which limits the statistical power and generalizability of the findings. As a result of this small sample size and the self‐paced structure of the submaximal cycling trial, we opted to analyze mean and peak values using paired *t*‐tests rather than performing repeated measures ANOVA on interval data. Although we collected data at each 10% increment of the submaximal test, the variable trial durations and pacing behaviors between subjects made alignment of time‐point data across participants inconsistent, potentially confounding group‐level interaction effects. Future studies with larger sample sizes and more standardized time‐aligned submaximal trials may benefit from incorporating a repeated measures ANOVA approach to further explore potential time × training interactions. There also may be differences in the impact of RMT on exercise performance in males versus females due to known sex‐specific differences in respiratory physiology. For instance, females typically exhibit smaller airway diameters, lower respiratory muscle strength, and a greater propensity for expiratory flow limitation during exercise, all of which may influence the Wb and responsiveness to RMT. Therefore, further studies should investigate whether these factors alter the perceptual or physiological adaptations to RMT in female participants. Additionally, the study did not have a sham intervention control group, which makes it difficult to determine whether the improvements observed in exercise performance were solely due to the RMT intervention. This investigation also did not examine the potential adverse effects of RMT, such as respiratory muscle fatigue, which may limit the practical application of RMT in some patient populations. Also worth noting, the increase in the V̇E/V̇CO_2_ ratio during the cycling trial following RMT is an intriguing finding, as it contrasts with the other positive outcomes of this study. Typically, a lower V̇E/V̇CO_2_ indicates more efficient gas exchange and reduced ventilatory effort. The increase observed here is somewhat unexpected, as a higher V̇E/VCO_2_ typically indicates reduced ventilatory efficiency. While the significance of this change is unclear, it may reflect subtle alterations in ventilatory control or metabolic response to exercise following RMT. Given that respiratory muscle strength was not directly measured, we refrain from attributing this outcome to enhanced respiratory function and suggest that future work explore this phenomenon in more detail.

Because our analysis was limited to end‐point values, we cannot draw conclusions about changes across the duration of the submaximal test. We therefore interpret our findings cautiously, noting that RMT did not alter overall trial performance but was associated with a higher HR at a fixed RPE. Larger studies with standardized, time‐aligned sampling are needed to assess whether RMT influences responses throughout exercise in hypoxia.

Finally, whether the RMT protocol utilized herein induced any changes in respiratory muscle function remains unknown due to the lack of any measurements associated with this training modality. Specifically, measurements of peak inspiratory flow, maximal inspiratory pressure, and ultrasound measurements of structural changes in the diaphragm would help support the notion that RMT is accomplishing the intended goals.

## 5 CONCLUSIONS

This is the first investigation to assess the impact of 4 weeks of endurance RMT on self‐paced exercise performance and V̇O_2_max in hypobaric hypoxia. Contrary to our original hypothesis, RMT did not significantly alter submaximal or maximal exercise performance. However, RMT did increase V̇O_2_max peak power and altered the HR‐RPE relationship during the submax‐CT. These results suggest that acute RMT may reduce the perception of fatigue during exercise in hypoxia, which could enhance performance. However, future investigations should assess the effects of RMT in larger and more diverse populations and explore the mechanisms underlying the observed improvements in exercise performance.

## CONFLICT OF INTEREST STATEMENT

The authors declare no conflicts of interest.

## Data Availability

The datasets generated during and/or analyzed during the current study are not publicly available due to participant confidentiality but are available from the corresponding author on reasonable request.
